# Some Facts about Feeble-Minded Children, and Their Care and Training in State Institutions

**Published:** 1883-03

**Authors:** Isaac N. Kerlin

**Affiliations:** Elwyn, Penn.


ATLANTA

### MEDICAL REGISTER.

VOL. II.] MARCH, 1883. [No. 6

### Original.

### SOME FACTS ABOUT FEEBLE-MINDED CHILDREN, AND THEIR CARE AND TRAINING IN STATE INSTITUTIONS.

BY ISAAC N. KERLIN, M.D., ELWYN, PENN.

According to the census of 1880, the population of the State of Georgia is 1,539,048—the twelfth State in the Union in point of population. In her improved acreage, she stands the tenth; and were the ratio struck on average breadths of territory, her position would be still higher in the scale.

The development of her manufacturing and mercantile interests has been astonishing, and in all that pertains to material prosperity, her growth is all that the most ardent son of Georgia could desire.

But her census develops also darker facts, which the humanitarian must study under the same light by which he traces the growth of a Commonwealth in the value of its acres, the activity of its mills and the thrift of its people.

Among every 233 of the population of Georgia stands one defective member, who is often an impediment and burden to the family and community, and always a sign of sorrow, wherever found.

Within the State of Georgia there are 1,699 insane, 1,634 blind, 819 deaf mutes, and 2,447 feeble-minded and idiotic children; or nearly as many feeble-minded and idiotic as of the deaf mutes and blind together.

It is the purpose of this article to bring before the reflecting members of the medical profession of Georgia the broad claim of the idiotic and feeble-minded population of the State, for recognition in her halls of legislation, and for a share of public and individual charity which should cover all human infirmity and deficiency; and especially strong is that claim of the dependent idiot or feeble-minded child, for he is so through no fault of his own. He is sinless, and yet the poor cross-bearer too often of others' sins. As Simon, touched with the master's degradation and broken body, hastened to lift a little of the heavy burden, so should we see the innocent cross-bearers of our own infirmities in these sad creatures, and to our utmost lighten their burdens. Under the blessing of God, this is being done in nearly every enlightened nation.

We know not how early the first attempts were made to comprehend and alleviate idiocy. The law or custom of many nations destroyed idiots in common with all disabled members of society. Later we find many instances of parental abandonment of such children.

The instinct of self-preservation drove them to the woods, where they led the lives of wild beasts, exposed on the mountains, and feeding on leaves and nuts, until, in a few years, they so completely lost the semblance of humanity that actually Linnaeus groups them as forming distinct varieties of our species, naming them after the beasts with which they were found living.

This ancient practice of abandonment, this terrible illustration of "man's inhumanity to man," is offset in the annals of other nations and tribes by the most remarkable devotion to the unfortunate; that house was blessed of God in which an imbecile was born; we read of the great Tycho Broke as having for his study companion an idiot, whose mutterings the astronomer listened to as for revelation; he shared the popular ideas of his time and neighborhood, that these creatures only walked on earth, their sight being in heaven; and in many parts of Ireland, Brittany, and among some of the lower classes of our own country, this same extravagance prevails. They are now habitually called "innocents" in Ireland and Brittany.

The term Cretin, applied to some of the worst forms of idiocy found in Alpine countries, is only a corruption of the name "Christian." They were called Chretiens by the peasantry of Switzerland, to imply that they are *par excellence*, children of the good God, irresponsible and incapable of displeasing him.

The first recorded experiment of training idiots and feeble-minded persons as educable beings is by a good old monk, St. Vincent de Paul, about 1633, whose labor in establishing foundlings and other hospitals was the little candle that shown through that dark and selfish age. He gathered into his sympathies a school of defective children at his priory of St. Lazarus; little more than the fact of it is now known.

It remained for two physicians of Paris—Itard and Seguin—between 1800 and 1843, to formally study the entire subject of idiocy and to devise scientific measures for its relief. Itard began with one of the sad cases brought to and classified by Linnaeus, and called by him *Juvenis Lnpinus*, or the wild boy of the Averyon.

He was described "as unused to our food—selecting his aliment by the smell, lying flat on the ground and immersing his chin in the water to drink, tearing all sorts of clothes, and trying constantly to escape, walking on all-fours, fighting with his teeth, giving but few marks of intelligence, having no articulate language, and devoid of the natural faculty of speech."

This unpromising object was to be educated out of his savagery by Itard to prove a certain theory of mental development then current among the scientists. He did not know that it was an abandoned idiot that he had to deal with. After years of toil spent upon him, he left his results which were satisfactory if his starting point had been understood. Seguin took up the wearisome task, established a school in Paris, and wrote a wonderful book on the "Principles of the Treatment of Idiocy."

What Seguin began in Paris had grown under that kind man's eye and directing hand until the week before his death, in 1881, he might have traveled among the nations of Europe, and into ten or twelve American States, and have found a hospitable welcome in their many public and private institutions devoted to his work.

The social position of a century and its growth in civilization and culture are known by its charitable institutions.

A few years ago, only, Australia was almost a blank on our school atlases; we were astonished that she occupied a proud place in our late Centennial Exposition, but her exhibit, impressive as it was, will not demonstrate her advance in prosperity so much as the fact that she has established an excellent institution for her idiotic and feeble-minded children, which now shelters more than 300 inmates. In the United States the first institution was opened at Barre, Mass., by Dr. H. B. Wilbur, in July, 1848—34 years ago. There are now not less than sixteen institutions for the training of feeble-minded children in this country. The following table will show the number of institutions already organized in the United States:

This article is conceived, and issued through the Atlanta Medical Register, with the sanguine hope that the medical profession, with its large social and public influence, will take an immediate lead in the proud work of furnishing in Georgia, a State Institution, for her idiotic and feeble-minded children.

It would be a painful inquiry to examine into the condition of these children as now scattered through the commonwealth, suffice it here to say that even wealth directed by the best intentions cannot often provide in the household the sympathy, even discipline, and cheerful variety these children need; they become sullen, obstinate, perhaps violent; they break all regulations that control the sane children of the family, and it is soon found a most serious, nay, impossible undertaking to rear a group of little ones wisely, with this mournful exception to all rule, and all inducement in their midst. Wealth either secretes the solitary one, brooding over it with melancholy tenderness, or places it permanently in the school or asylum of another State, that it may there enjoy all the light and development possible to its dwarfed capacities.

Wealth, with intelligence and sympathy directing, protects its own local community, the family and neighhood—by thus interposing in one way or another, a kindly barrier between the afflicted one and those whom he would injure or be injured by.

And it is thus that the State and community, wealthy in their resources, will do by the feeble minded-child of the laborer and the mechanic—are not all these forms of broken humanity the natural wards of the strong and benevolent? When intelligence and sympathy shall direct the charity of the willing and the legislation of the State.

In the organization of this new work for Georgia, there should be no illusory promises made. Idiocy and most of its unfortunate concomitants, are too often radical infirmities not susceptible of cure, but conditions for help and amelioration always. The query, what proportion of the feeble minded under training, become self-supporting, is often made and after a long experience, I answer: it is certain that of those who are sent out from institutions of the kind as self-supporting there are but few individuals who will not always need judicious and considerate guardianship; they lack that judgment or forecast which anticipates and provides for the needs of the future—they possess little or no insight of character—they are either irritable and suspicious or weakly credulous, lacking that combativeness which is self-protection and which gives equality among fellows; hence without the guardianship of merciful relations or friends, who are considerate of their defects, they fail of success, are bitterly imposed upon, or may become the easy dupes and facile tools of rascals and knaves. Of 1000 individuals received and trained at the Pennsylvania Institution, 162 are capable of earning their own support in domestic service, farming, or certain shop employments, under influences of favorable protection, such as have been named; 280 are too uncertain for a real dependence, and yet may be rated as capable of earning a half support; 136 perform very small services of no appreciable value in themselves; 321 are hopelessly and totally dependent, earning nothing. We note their developing attention to personal wants, their improvement in delicacy, language, or movement, as the only evident testimony to the offices of kindness, forbearance and teaching which they receive. Our chief encouragement in this department of our work must come in the warm expressions of relieved homes, and from the convictions in our own hearts that unto even these "little ones" the "cup of cold water" cannot be denied.

The reader will note that we claim 16 per centum as being brought to a state of self support—under certain helpful conditions. This is the best we can claim for our work in Pennsylvania. The Ohio Institution has claimed as high as 24 per centum, which is due probably to the superior facilities that institution enjoys for teaching farming and gardening, and possibly more to the fact, that mental weakness west of the Alleghanies, or in newer communities is associated less with paralysis, epilepsy and other neurotic complications than on the seaboard or in older communities. Another frequent inquiry, suggested by one who doubts the utility of our work is "Do they relapse into their former condition when discharged from the institution?"

Yes, if they return to homes where no rational control exists, or are thrown into alms houses and asylums for insane, they may relapse; they certainly will relapse if uncurbed and uncared for, if they are exposed to the vice ignorance and vagrancy of the courts and alleys of our great cities. They do not relapse when passing from the Institution into proper homes and discreet guardianship.

A child of school grade—that is a feeble-minded child of middle or upper grade—well trained during five or seven years or even for less periods, will not only retain the good and gain which have been made, but will go on improving, provided a few of his new surroundings are of a character to induce and not cripple such advancement. And here is where we have met discouragement. Some of the most worthy parents are entirely inadequate to that temperate, kindly and yet firm control which such cases demand. A few have no asylum or protection at home when compelled to leave us, and are left to most corrupt and contaminating influences. Very many are orphans, who, unless kindly taken up, as is seldom the case, by relatives, or their work so remunerative as to make them desirable servants, must drop into the maelstrom of pauperism, their lives perhaps a little brighter, and retaining a little more the semblance and feelings of our humanity than could have been the case without such favorable teachings upon their developing years as the public institution furnishes. The idea that an improved imbecile or idiot is only made more unhappy from an awakened consciousness of his infirmity, or when remanded to an alms house, so much the more miserable, because of what he had enjoyed in the public Institution, is not sustained in fact.

We have traced some of our discharged children into the unfortunate surroundings of the alms house and insane asylum, and found them warmly commended by the officers for their great improvement in obedience, cleanliness, willingness, and capacity to work. But when we can follow them back into the orderly surrounding of a good home, be it even an humble one, we find the richest rewards for our labor, and the warmest advocates for the Institution.

Perhaps we have here indicated the class in the community for which the State provision at first is best extended—the provident poor and middle or mechanic classes; help for those who are bravely trying to help themselves.

In the office of Samuel Sloan, the veteran architect of Philadelphia, hangs a large and beautiful picture of an extensive and tastefully planned edifice. It suggests to the eye the sentiment of a great home school, and with its shaded porches, extending through the stories, it is, unmistakably, designed for a Southern site; and indeed its title reads "The Georgia Institute for Feeble-Minded Children."

As long ago as 1870 the question of such an institution was agitated by some leading citizens of your State, and the experienced architect was requested to draft on paper the best posssible conception of a training school for two hundred feeble-minded. It remains yet for the conception to receive its breath of life, to become potential for good to hundreds of the afflicted children of your commonwealth, and one of the State's brightest jewels.

